# Small interfering RNA-mediated suppression of Ccl2 in Müller cells attenuates microglial recruitment and photoreceptor death following retinal degeneration

**DOI:** 10.1186/1742-2094-9-221

**Published:** 2012-09-19

**Authors:** Matt Rutar, Riccardo Natoli, Jan M Provis

**Affiliations:** 1 The John Curtin School of Medical Research, College of Medicine, Biology and Environment, The Australian National University, Building 131, Garran Rd, Canberra, ACT 0200, Australia; 2 ARC Centre of Excellence in Vision Science, The Australian National University, Canberra, ACT 0200, Australia; 3 ANU Medical School, The Australian National University, Canberra, ACT 0200, Australia

## Abstract

**Background:**

The recruitment and activation of inflammatory cells is thought to exacerbate photoreceptor death in retinal degenerative conditions such as age-related macular degeneration (AMD). We investigated the role of Müller cell-derived chemokine (C-C motif) ligand (Ccl)2 expression on monocyte/microglia infiltration and photoreceptor death in light-mediated retinal degeneration, using targeted small interfering (si)RNA.

**Methods:**

Adult Sprague–Dawley rats were injected intravitreally with 1 μg of either Ccl2 siRNA or scrambled siRNA, and were then exposed to 1000 lux of light for a period of 24 hours. The mice were given an overdose of barbiturate, and the retinas harvested and evaluated for the effects of bright-light exposure. Ccl2 expression was assessed by quantitative PCR, immunohistochemistry, and *in situ* hybridization. Monocytes/microglia were counted on retinal cryostat sections immunolabeled with the markers ED1 and ionized calcium binding adaptor (IBA)1, and photoreceptor apoptosis was assessed using terminal dUTP nick end labeling.

**Results:**

Intravitreal injection of Ccl2 siRNA significantly reduced the expression of Ccl2 following light damage to 29% compared with controls. In retinas injected with Ccl2 siRNA, *in situ* hybridization and immunohistochemistry on retinal cryostat sections showed a substantial decrease in Ccl2 within Müller cells. Cell counts showed significantly fewer ED1-positive and IBA1-positive cells in the retinal vasculature and outer nuclear layer of Ccl2 siRNA-injected retinas, compared with controls. Moreover, there was significantly less photoreceptor apoptosis in Ccl2 siRNA-injected retinas compared with controls.

**Conclusions:**

Our data indicate that Ccl2 expression by Müller cells promotes the infiltration of monocytes/microglia, thereby contributing to the neuroinflammatory response and photoreceptor death following retinal injury. Modulation of exaggerated chemokine responses using siRNA may have value in reducing inflammation-mediated cell death in retinal degenerative disease such as AMD.

## Background

Microglial cells are a major retinal glial constituent derived from the mononuclear phagocyte lineage, and play a crucial role as the principle resident immunocompetent and phagocytic cells of the central nervous system (CNS), including the retina. Through persistent surveillance of their microenvironment, microglia act as motile sensors that help maintain homeostasis in retina through a variety of functions, including facilitating phagocytosis of debris and apoptotic cells [1-3], antigen presentation [4-7], and secretion of neuroprotective factors [8,9].

Recruitment of microglia/monocytes to damaged regions occurs in almost every pathological condition in the CNS [10,11], and is apparent in a range of prominent human retinal pathologies including age-related macular degeneration (AMD) [2,12-15], retinitis pigmentosa [2], late-onset retinal degeneration [2], retinal detachment [16], glaucoma [17-19], and diabetic retinopathy [17,20], as well as in many experimental models of retinal degeneration [9]. Despite their beneficial properties, widespread recruitment and activation of microglia may damage neurons [21-25], probably through their secretion of pro-inflammatory mediators and cytotoxic factors, such as tumor necrosis factor (TNF)-α, interleukin (IL)-1β [10,26,27], and nitric oxide [23,28,29]. Moreover, microglial activation is directly implicated in models of neovascular AMD [30], light-induced damage [21,31-33], diabetic retinopathy [34,35], glaucoma [36,37], chronic photoreceptor degeneration in *rds* (retinal degeneration slow) mice [38], and photoreceptor apoptosis *in vitro*[22].

In spite of their prominent role in retinal degeneration, the precise signaling events that mediate the trafficking of microglia/monocytes in the retina are not yet elucidated [39]. Chemokines are a large family of molecules that have potent chemoattractant properties in the recruitment of leukocytes in immune surveillance and inflammation in the CNS [40-43]. Chemokine expression results in the establishment of chemical ligand gradients that serve as directional cues for the guidance of certain leukocytes to sites of injury [40]. Chemokine (C-C motif) ligand (Ccl)2 is one of the most well-characterized chemokines [44], and is a potent chemoattractant and activator for monocytes and microglia *in vitro*[45-47]. Upregulation of Ccl2 is also implicated in a number of CNS pathologies such as Alzheimer’s disease [48,49], multiple sclerosis [50,51], frontotemporal lobe dementia [52], and brain trauma [53,54]. We have shown previously that the expression of Ccl2 is upregulated in Müller cells in a light-induced model of retinal degeneration [55], which coincides spatiotemporally with the local recruitment of microglia/monocytes and the region of peak photoreceptor death [56].

In the current study, we aimed to investigate the role of Müller-cell-derived Ccl2 in the recruitment of retinal monocytes/microglia following exposure to bright continuous light (BCL), using targeted small interfering (si)RNA to suppress Ccl2 expression in the retina. siRNA molecules are short sequences of double-stranded RNA, which serve as a component of RNA interference (RNAi) [57]. The RNAi cellular machinery enables the specific degradation of a target mRNA of complementary sequence, which effectively silences expression of the particular gene [58]. In this study, we found that intravitreal administration of Ccl2 siRNA suppressed expression of Ccl2 by Müller cells, resulting in an inhibition of microglia/monocyte recruitment and reduction in photoreceptor death following BCL exposure. Consequently, inhibition of endogenous chemokine expression using siRNA may present a viable means to modulate excessive microglial activation in the degenerating retina.

## Methods

### Ethics approval

The study was approved by the Animal Experimentation Ethics Committee (AEEC) of the Australian National University (R.BSB.05.10). All experiments conducted were in accordance with the Association for Research in Vision and Ophthalmology Statement for the Use of Animals in Ophthalmic and Vision Research.

### Animals

Adult Sprague–Dawley (SD) rats aged between postnatal days 160 and 190 were used for the experiments. The rats were born and reared in dim cyclic light conditions with an ambient level of approximately 5 lux, until the commencement of bright-light exposure.

### Preparation of small interfering RNA and intravitreal injection

RNAi was achieved using a cocktail of two commercially available modified siRNAs, specific for Ccl2 (Stealth siRNA; #RSS302703 and #RSS302704; Invitrogen Inc., Carlsbad, CA USA). A scrambled siRNA equivalent, not homologous to any known gene, served as a negative control, and was conjugated to an Alexa 555 fluorophore to assess the uptake of siRNA in the retina (#14750–100; Invitrogen Inc.). Before administration, siRNAs were encapsulated using a cationic liposome-based formulation (Invivofectamine; #1377–901; Invitrogen Inc.) in accordance with the manufacturer’s instructions. The final concentration of each encapsulated siRNA formulation was 0.33 μg/μl in a 5% glucose solution.

For intravitreal injections, animals were anaesthetized with an intraperitoneal injection containing 30 mg ketamine (100 mg/ml; Troy Laboratories, NSW, Australia) and 3 mg xylazil (20 mg/ml; Parnell, NSW, Australia). A drop of 1% atropine (Chauvin Pharmaceuticals, London, England) was applied to the ocular surface to produce mydriasis, and the injection site was then swabbed with 5% povidone iodine (Betadine; Faulding Pharmaceuticals, SA, Australia). Intravitreal injection was then performed as described previously [59]; 3 μL of either positive or negative siRNA complex, corresponding to 1 μg of siRNA, was injected into both eyes of each animal. For an additional control, 3 μLof transfection agent only was also injected into both eyes of some animals. After injection, neomycin ointment 5 mg/g (Amacin; Jurox, NSW, Australia) was applied to the injection site to prevent infection.

### Light exposure

After intravitreal injections, the animals were immediately transferred to individual cages designed to allow light to enter unimpeded. BCL exposure was achieved using an 18 W fluorescent light source (Cool White; TFC, Taipei, Taiwan) positioned above the cages, , which was run from 11.00 to 24.00 hours, and kept at an intensity of approximately 1000 lux at the cage floor. Corneal hydration was maintained by application of a synthetic tear gel (GenTeal Gel; Novartis, NSW, Australia) during BCL, until the animals awoke. Animals were exposed to BCL for 24 hours before tissue collection.

### Tissue collection and processing

Animals were killed using an overdose (60 mg/kg bodyweight) of barbiturate (Valabarb; Virbac, Australia) given as intraperitoneal injection, then retinal tissue was obtained from each treatment group for analysis. Eyes from some animals were marked at the superior surface for orientation, then enucleated and processed for sectioning on a cryostat, while the retina from others was excised through a corneal incision and prepared for RNA extraction.

Eyes for sectioning were immediately immersion-fixed in 4% paraformaldehyde in 0.1 M PBS (pH 7.3) for 3 hours at room temperature, then processed as previously described [56], and sectioned at 16 μm on a cryostat. Retinas for RNA extraction were immediately immersed in chilled solution (RNAlater; #7024; Ambion Inc., Austin, TX, USA), then stored in accordance with the manufacturer’s instructions. The RNA was then extracted from each sample and analyzed as previously described [55,60].

### Quantitative real-time PCR

First-strand cDNA synthesis was performed as described previously [55]. Gene amplification was measured using commercially available hydrolysis probes (TaqMan®; Applied Biosystems, Foster City, CA, USA) (Table 1). The hydrolysis probes were used in accordance with a previously described quantitative (q)PCR protocol [55]. The fold change was analyzed using the ^ΔΔ^C_q_ method, with expression of the target gene normalized relative to the expression of two reference genes: glyceraldehyde-3-phosphate dehydrogenase (GAPDH), and β-actin. Amplification specificity was assessed using gel electrophoresis. 

**Table 1:** Taqman probes used

**Gene symbol**	**Gene name**	**Catalog number**	**Entrez Gene ID number**
β-actin	Beta-actin	Rn00667869_m1	NM_031144.2
Ccl2	Chemokine (C-C motif) ligand 2	Rn01456716_g1	NM_031530.1
GAPDH	Glyceraldehyde-3-phosphate dehydrogenase	Rn99999916_s1	NM_017008.3
Jun (AP-1)	Jun oncogene (transcription factor activator protein −1)	Rn99999045_s1	NM_021835.3

### *In situ* hybridization

To investigate localization of Ccl2 mRNA transcripts in the retina following RNAi, a riboprobe to Ccl2 was generated for *in situ* hybridization on frozen sections of retinal tissue. Synthesis of the Ccl2 riboprobe and *in situ* hybridization were performed as described previously [55,61]. The Ccl2 riboprobe was hybridized overnight at 55°C, and then washed in saline sodium citrate (pH 7.4) at 60°C. After hybridization, some sections were further stained using immunohistochemistry (see below).

### Analysis of cell death

Following BCL, terminal dUTP nick end labeling (TUNEL) was used to quantify photoreceptor apoptosis in cryostat sections for each treatment group, using a previously published protocol [62]. Counts of TUNEL-positive cells in the outer nuclear layer (ONL) were carried out along the full length of retinal sections cut in the parasagittal plane (superio-inferior), within the vertical meridian. The total count from each retina is the average of four sections at comparable locations.

### Immunohistochemistry

Frozen sections from each treatment group were used for immunohistochemical analysis, using the primary antibodies listed in Table 2. Immunohistochemistry was performed as previously described [55]. Immunofluorescence was viewed using a laser scanning microscope (Carl Zeiss, Jena, Germany), and acquired using PASCAL software (version 4.0; Carl Zeiss). Images were prepared for publication using Adobe Photoshop software. 

**Table 2:** Antibodies used for immunohistochemistry

**Antibody**	**Dilution**	**Source**	
		**Catalog number**	**Manufacturer**
Hamster α-Ccl2	1:100	505902	Biolegend, San Diego, CA, USA
Mouse α-ED1	1:200	MAB1435	Invitrogen Inc., Carlsbad, CA, USA
Rabbit α-IBA1	1:400	019–19741	Wako, Osaka, Japan
Mouse α-S100β	1:200	S2532	Sigma Chemical Co., St. Louis, MO, USA
Mouse α-vimentin	1:200	18-0052	Zymed, San Francisco, CA, USA

CCL, chemokine (C-C motif) ligand; IBA, ionized calcium binding adaptor.

### Quantification of monocytes/microglia

Monocyte/microglia counts were performed on sections immunolabeled jointly with the markers ED1 and ionized calcium binding adaptor (IBA)1. Numbers of ED1+/IBA + and ED1−/IBA1+ nuclei were assessed long the full length of retinal sections cut in the parasagittal plane (supero-inferior) within the vertical meridian. Counts were made of all ED1+/IBA + monocytes throughout the retina, including the retinal vasculature, ONL, and choroidal vasculature. Counts of /ED1−/IBA1+ parenchymal microglia encompassed those in the outer plexiform layer (OPL) and ONL/subretinal space (but not the resting population in the inner plexiform layer; IPL), as microglia recruit to these areas when activated during retinal degeneration [9,47,63]. The total counts of ED1+/IBA + and ED1−/IBA1+ nuclei from each retina was the average of four sections at comparable locations.

### Quantification of chemokine (C-C motif) ligand (Ccl)2-expressing Müller cells

Ccl2 expression following RNAi in Müller cells was assessed on frozen sections after either immunohistochemistry or *in situ* hybridization for Ccl2 (as described above). In Ccl2-immunolabeled sections, the number of Ccl2-immunoreactive Müller cell processes was assessed. In sections used for *in situ* hybridization, counts were made of Ccl2-expressing Müller cell bodies. Both sets of counts were conducted across the full length of retinal sections cut in the parasagittal plane (supero-inferior) within the vertical meridian; the total count was the average of four sections at comparable locations.

### Statistical analysis

Statistical analysis for each experiment was performed using one-way ANOVA with Tukey’s multiple comparison *post hoc* test. For each analysis, *P* < 0.05 was considered significant.

## Results

### Localization of transfected small interfering RNA in the retina

**Figure 1:**
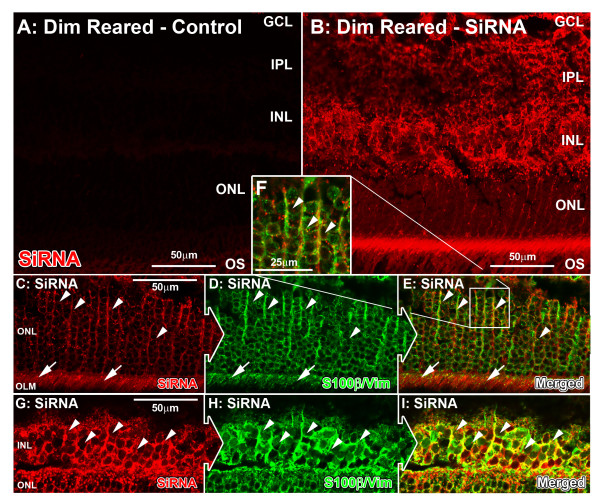
**Localization of fluorescently-tagged small interfering (si)RNA following intravitreal injection in control retinas. (A)** Fluorescence was not detected in retinas injected with Invivofectamine only in mice reared in dim light. **(B)** Strong fluorescence for injected siRNA (red) was seen after 24 hours of incubation, which was apparent throughout all cellular layers through to the outer nuclear layer (ONL). **(C-I)** Dual immunolabeling of siRNA (red) and the Müller-cell-specific proteins S100β/vimentin (green) showed colocalization of siRNA with S100β/vimentin in Müller cell processes situated within the **(C–F)** ONL and outer limiting membrane (OLM) (arrows), and **(G–I)** the inner nuclear layer (INL) (arrows). GCL, ganglion cell layer; IPL, inner plexiform layer; OS, outer segments.

To assess the efficacy of the siRNA transfection protocol, animals reared in dim light conditions were injected intravitreally with siRNA tagged with Alexa 555 to determine the cellular uptake of siRNA in the retina (Figure 1). At 24 hours after injection of the fluorophore-tagged siRNA, fluorescence for siRNA was visible deep within the retinal cellular layers, including transfection in the ganglion cell layer (GCL), inner nuclear layer (INL), and ONL (Figure 1B). Control animals who had not been injected with the fluorophore-tagged siRNA had no comparative fluorescence (Figure 1A). Using fluorescent markers, the transfected siRNA also showed colocalization with vimentin-immunoreactive Müller cell processes within the INL (Figure 1G-I; arrows), ONL, and outer limiting membrane (Figure 1C-E; arrows).

### Suppression of chemokine (C-C motif) ligand (Ccl)2 expression with small interfering RNA following light damage

**Figure 2:**
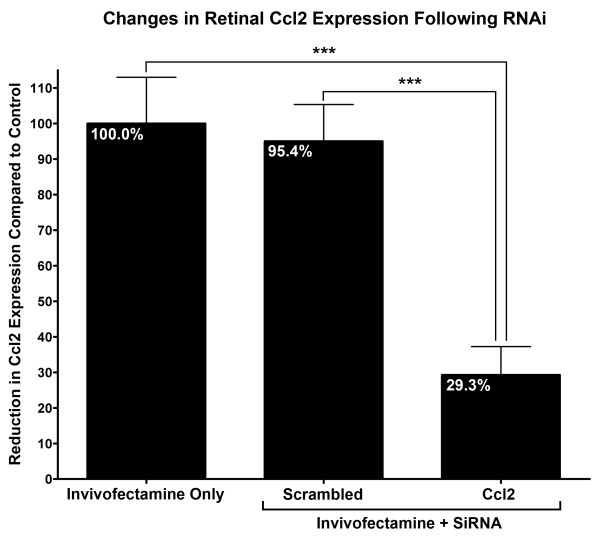
**Relative expression of Ccl2 in the retina measure by quantitative PCR following small interfering (si)RNA injection and light damage.** In animals injected with Ccl2 siRNA, expression of Ccl2 decreased significantly to 29.3% relative to the Invivofectamine-only control group after 24 hours BCL (*P* < 0.05; ANOVA/Tukey’s test), whereas expression of Ccl2 in animals injected with scrambled siRNA did not change appreciably compared with the Invivofectamine-only group (95.4%, *P* < 0.05; ANOVA/Tukey’s test). Inivofectamine-only (n = 8), scrambled siRNA (n = 8), Ccl2 siRNA (n = 6). Error bars represent SEM. *Significant change at *P* <0.05 using ANOVA with Tukey’s *post hoc* test.

Retinal expression of Ccl2 following Ccl2 siRNA injection was assessed using qPCR (Figure 2). In animals injected with Ccl2 siRNA, expression of Ccl2 decreased significantly to 29.3% (*P* < 0.05; ANOVA/Tukey’s test) of that in retinas injected with Invivofectamine after 24 hours of BCL. Expression of Ccl2 in retinas injected with scrambled siRNA did not change appreciably, remaining at 95.4% if of that of the Invivofectamine-only controls (95.4%, *P* > 0.05; ANOVA/Tukey’s test).

**Figure 3:**
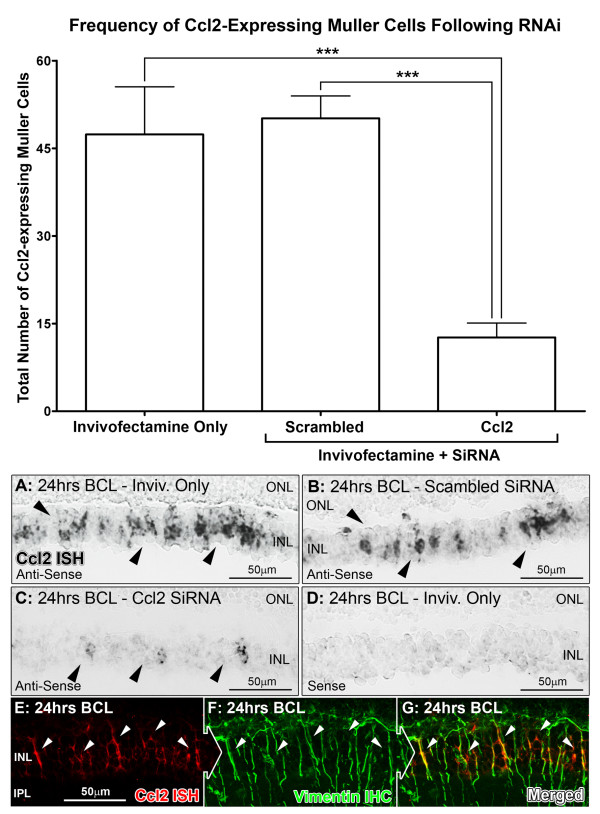
**Chemokine (C-C motif) ligand (Ccl)2 expression in M**ü**ller cells following Ccl2 small interfering (si)RNA treatment and light damage.** (**A**–**C**) Representative images taken from the superior mid-periphery showing *in situ* hybridization results for Ccl2 mRNA within processes situated in the inner nuclear layer (INL) following exposure to bright continuous light (BCL; black arrows). **(D)** Sense controls showed no specific staining. **(E–G)** Co-labelling for Ccl2 mRNA with a fluorescent stain (red) showed colocalization for vimentin-immunoreactive (green) Müller cell processes (white arrows). The histogram shows that the number of Ccl2-expressing Müller cells per retina decreased significantly in the Ccl2 siRNA-treated group (12.6 cells) compared with the Invivofectamine-only (47.4 cells; *P* < 0.05) and the scrambled siRNA (50.1 cells; *P* < 0.05) groups. Inivofectamine-only (n = 4), scrambled siRNA (n = 4), Ccl2 siRNA (n = 4). Error bars represent SEM. *Significant change at *P* <0.05 using ANOVA with Tukey’s *post hoc* test. ONL, outer nuclear layer.

**Figure 4:**
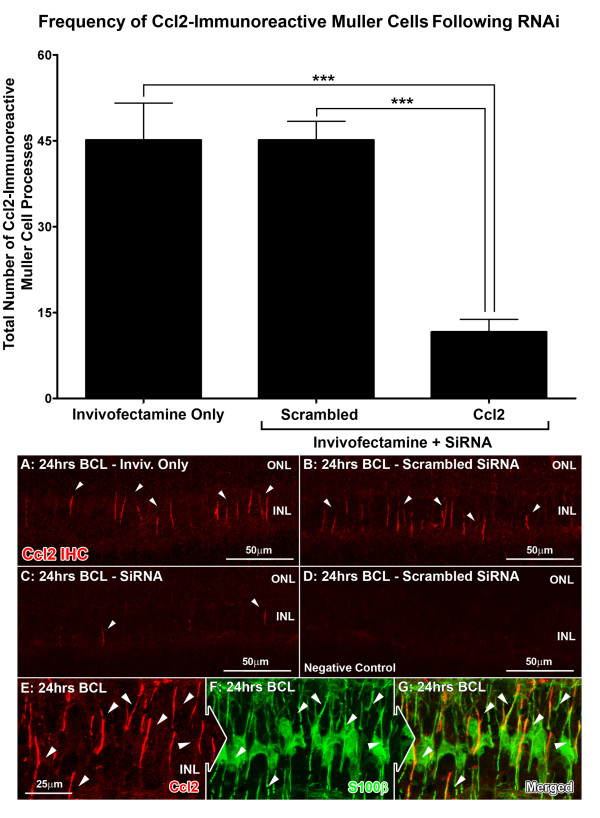
**Immunoreactivity (IR) for chemokine (C-C motif) ligand (Ccl)2 protein in M**ü**ller cells following light damage in relation to Ccl2 small interfering (si)RNA treatment.** (**A**–**D**) Representative images from the superior mid-periphery showed strong IR for Ccl2 (red) in radially oriented processes within the inner nuclear layer (INL) following **(A–C)** BCL (white arrows), whereas **(D)** negative controls showed no fluorescence. **(E–G)** The Ccl2 IR showed strong colocalization for S100B-immunoreactive (green) Müller cell processes (white arrows). The histogram of the quantification of Ccl2-IR Müller cell processes per retina indicated a substantial reduction in animals injected with Ccl2 siRNA (11.6, *P* < 0.05) compared with those injected with Invivofectamine only or with scrambled siRNAs (45.1 and 45.2 respectively). Inivofectamine-only (n = 4), scrambled siRNA (n = 4), Ccl2 siRNA (n = 4) Error bars represent SEM. *Significant change at *P* <0.05 using ANOVA with Tukey’s *post hoc* test. ONL, outer nuclear layer.

Localization of Ccl2 mRNA and protein following 24 hours of BCL was assessed in retinas using *in situ* hybridization (Figure 3) and immunoreactivity (IR) (Figure 4) respectively. The distribution of both Ccl2 mRNA and protein following BCL showed strong colocalization for, respectively, vimentin-immunoreactive (Figure 3E-G, arrows) and S100B-immunoreactive (Figure 4E-G, arrows) Müller cell processes, consistent with our previous investigation [55]. After Ccl2 siRNA injection, the number of Müller cells expressing Ccl2 mRNA decreased significantly to 12.6 per retina, compared with 47.4 per retina in Invivofectamine-only controls (*P* < 0.05, ANOVA-Tukey’s test) (Figure 3). Scrambled siRNA-injected retinas showed no significant change in the number of Ccl2-expressing Müller cells, compared with Invivofectamine-only controls (50.1 per retina, *P* > 0.05; ANOVA/Tukey’s test). IR for Ccl2 protein (Figure 4 histogram) showed a significant reduction in the number of Ccl2-IR Müller cell processes in retinas injected with Ccl2 siRNA (11.6 per retina, *P* < 0.05; ANOVA/Tukey’s test) compared with retinas treated with Invivofectamine only or with scrambled siRNA (45.1 and 45.2 per retina, respectively). 

### Quantification of monocyte/microglia recruitment after Ccl2 siRNA injections

**Figure 5:**
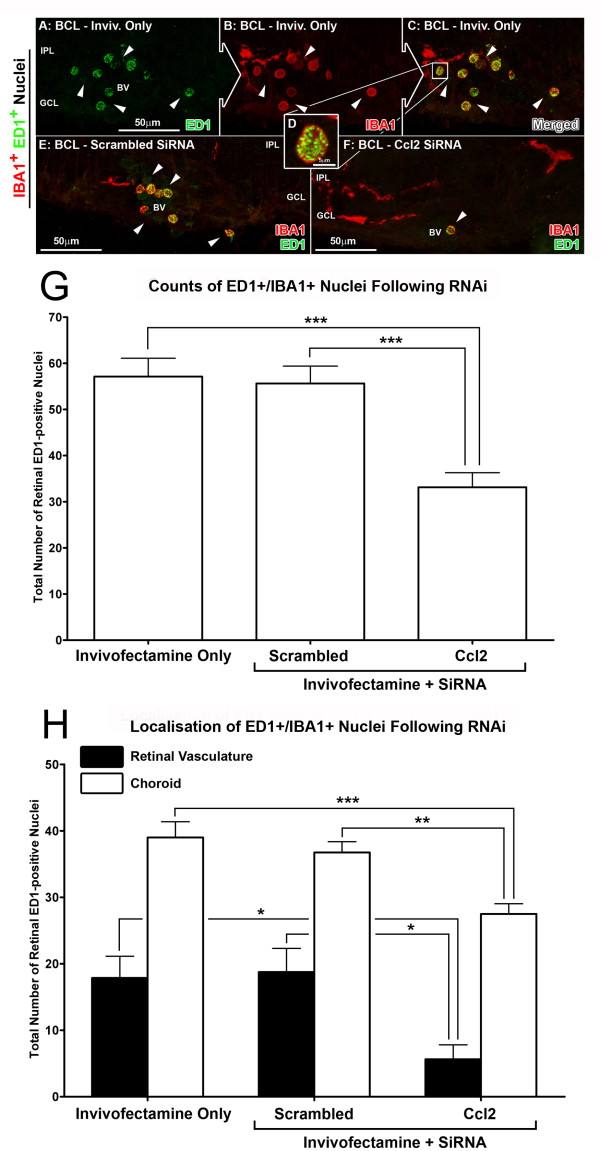
**Recruitment of monocytes positive for ED1 and ionized calcium binding adaptor molecule (IBA)1 following treatment with bright continuous light (BCL) in retinas injected with chemokine (C-C motif) ligand (Ccl)2 small interfering (si)RNA. (A-F)** Representative images taken from the superior mid-periphery showed immunoreactivity for ED1 (green) and IBA1 (red) in ED1+/IBA1+ nuclei recruited to the retinal vasculature following BCL (arrows). **(G)** The total number of these ED1+/IBA1+ nuceli per retina was significantly reduced in Ccl2 siRNA-injected retinas following BCL compared with the Invivofectamine-only group and the scrambled siRNA control group (*P* < 0.05). **(H)** Numbers of ED1+/IBA1+ nuclei recruited to retinal and choroidal vasculature locations were reduced in retinas injected with Ccl2 siRNA in comparison to controls (*P* < 0.05). Inivofectamine-only (n = 4), scrambled siRNA (n = 4), Ccl2 siRNA (n = 4) Error bars represent SEM. *Significant change at *P* <0.05 using ANOVA with Tukey’s *post hoc* test. GCL, ganglion cell layer; IPL, inner plexiform layer.

**Figure 6:**
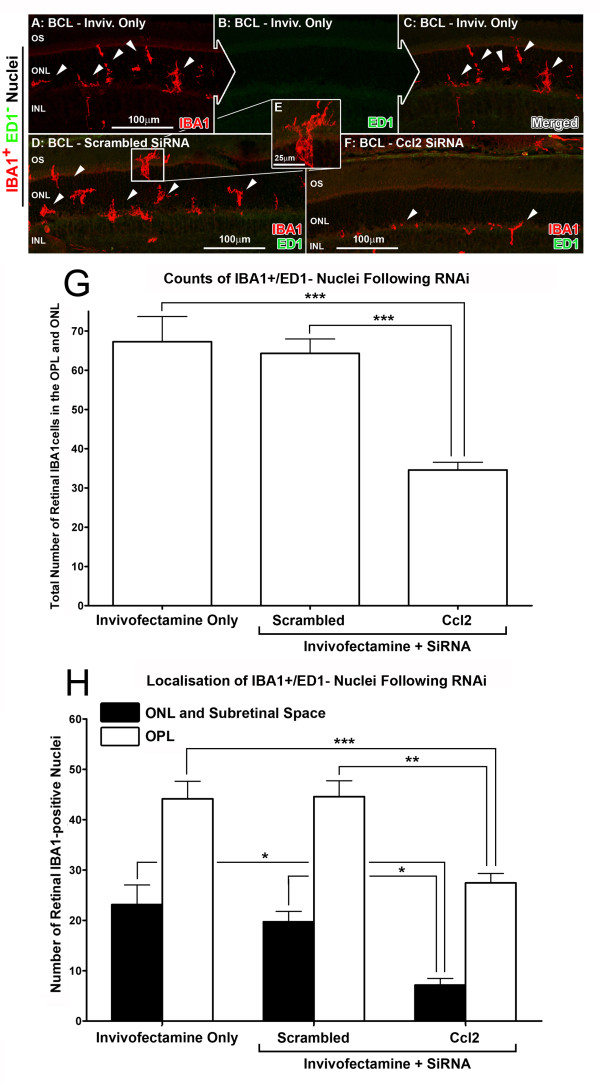
**Recruitment of microglia positive for ionized calcium binding adaptor molecule (IBA)1 and negative for ED1 following BCL in retinas injected with chemokine (C-C motif) ligand (Ccl)2 small interfering (si)RNA. (A–F)** Representative images from the superior mid-periphery showed immunoreactivity for IBA1 (red), but not ED1 (green), in ramified ED1−/IBA1+ nuclei that were recruited to the outer nuclear layer (ONL) and outer plexiform layer (OPL) after 24 hours of BCL (**A**–**C**; arrows). **(G)** The total number of these recruited ED1−/IBA1+ nuclei per retina decreased significantly in Ccl2 siRNA-injected retinas following BCL, compared with both the Invivofectamine-only group and the scrambled siRNA control group (*P* < 0.05). **(H)** Numbers of ED1−/IBA1+ nuclei recruited to either the OPL or the ONL/subretinal space were reduced in retinas injected with Ccl2 siRNA in comparison with control groups (*P* < 0.05). Inivofectamine-only (n = 4), scrambled siRNA (n = 4), Ccl2 siRNA (n = 4). Error bars represent SEM. *Significant change at *P* <0.05 using ANOVA with Tukey’s *post hoc* test. INL, inner nuclear layer; OS, outer segments.

The recruitment of monocytes/microglia in the retina following siRNA injections was assessed using immunolabeling for IBA1 and ED1 markers (Figures 5 and 6), which identify both monocytes (ED1+/IBA1+) and ramified microglia (ED1−/IBA1+) [64,65]. After BCL exposure, ED1+/IBA1+ nuclei (Figure 5A-C) were recruited to the retinal and choroidal vasculature (as described previously [55]). These nuclei were reduced significantly in total counts to 33.1 per retina in the Ccl2 siRNA group (*P* < 0.05; ANOVA-Tukey’s), compared with 57.1 and 55.6 per retina in the Invivofectamine-only and the scrambled siRNA groups respectively (Figure 5G). The numbers of recruited ED1+/IBA1+ cells in different locations (retinal vasculature, choroid) are shown in Figure 5H. These counts show a significant reduction in the recruitment of ED1+/IBA1+ nuclei to both the choroid and retinal vasculature in Ccl2 siRNA treated animals, compared with controls (*P* < 0.05; ANOVA-Tukey’s). ED1−/IBA1+ nuclei were recruited to the ONL and outer plexiform layer (OPL) after 24 hours BCL (Figure 6A-C). Following Ccl2 siRNA injection, the total number of these nuclei was found to decrease significantly to 34.6 per retina (*P* < 0.05; ANOVA-Tukey’s) in comparison to those injected with either Invivofectamine only or scrambled siRNA (67.3 and 64.3 per retina respectively, Figure 6G). The recruitment of ED1−/IBA1+ nuclei to both locations (OPL, and ONL/subretinal space) was reduced in siRNA-injected animals, compared with controls (*P* < 0.05; ANOVA/Tukey’s test, Figure 6B). 

### Assessment of apoptosis in the retina following suppression of Ccl2 with siRNA

**Figure 7:**
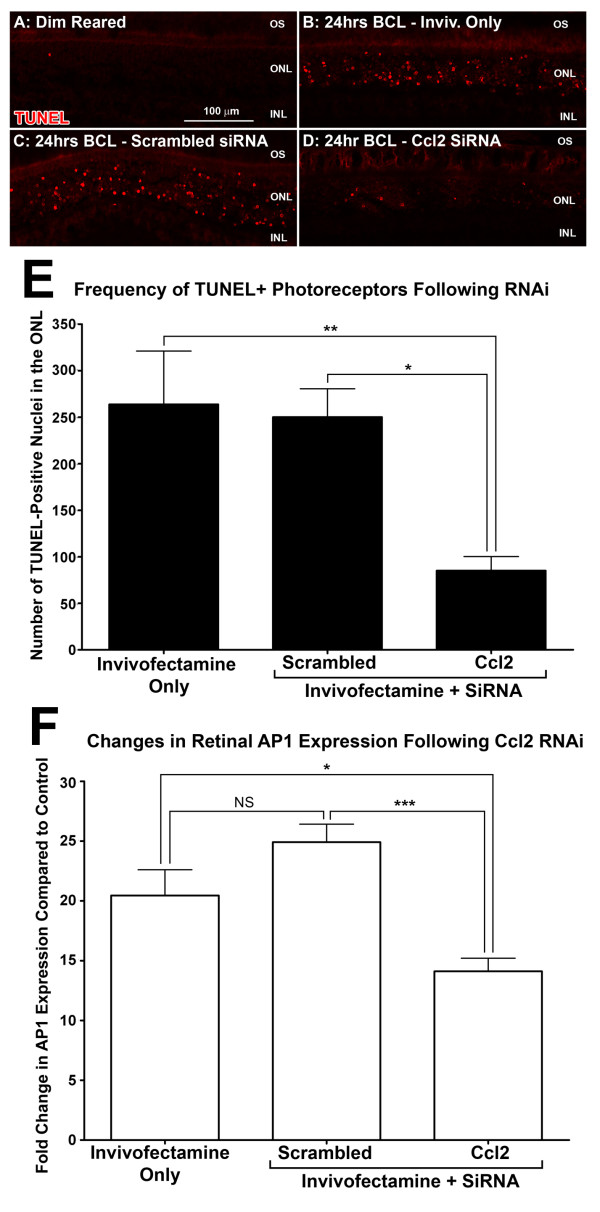
**Quantification of apoptosis following BCL by terminal dUTP nick end labeling (TUNEL) and activator protein (AP)-1 expression in retinas injected with chemokine (C-C motif) ligand (Ccl)2 small interfering (si)RNA. (A–D)** Representative images from the superior mid-periphery show TUNEL (red) for nuclei situated predominantly in the ONL in the siRNA treatment groups following BCL exposure. E: Animals injected with Ccl2 siRNA found a marked decrease in the number of TUNEL-positive nuclei in the outer nuclear layer (ONL) (85.1, *P* < 0.05; ANOVA/Tukey’s test) compared with the Invivofectamine-only group and the scrambled siRNA control group after 24 hours of BCL (263.8 and 250.1 respectively). **(F)** Expression of AP-1 in the retina following BCL was reduced to 14.1-fold in retinas injected with Ccl2 siRNA (*P* < 0.05), compared with 20.5-fold and 24.9-fold reduction in the Invivofectamine-only group and the scrambled siRNA control group, respectively. Inivofectamine-only (n = 8), scrambled siRNA (n = 8), Ccl2 siRNA (n = 6) Error bars represent SEM. *Significant change at *P* <0.05 using ANOVA with Tukey’s *post hoc* test. NS, not significant.

There was no significant change in the number of TUNEL-positive photoreceptors (Figure 7A-E,) seen throughout the retina following BCL between animals intravitreally injected with either Invivofectamine only or scrambled siRNA (263.8 and 250.1 per retina, *P* > 0.05; ANOVA/Tukey’s test). However, in animals injected with Ccl2 siRNA, a marked decrease in the number of TUNEL-positive photoreceptors (to 85.1 per retina) was seen after BCL compared with both the Invivofectamine-only group and the scrambled siRNA control group (*P* < 0.05; ANOVA/Tukey’s test). In conjunction, expression of the apoptosis-related gene Jun (activator protein-1) [66] following BCL (Figure 7B) was markedly reduced in animals injected with Ccl2 siRNA, compared with both control groups (*P* < 0.05; ANOVA/Tukey’s test). 

## Discussion

The findings of the current study confirm a key role for Müller cells and Ccl2 in the retinal neuroinflammatory response in the light-damage model of retinal degeneration. Firstly, using both *in situ* hybridization and immunohistochemistry, we confirmed the efficacy of siRNA transfection in targeted suppression of Ccl2 expression in Müller glia following damage. Second, we found that suppression of Ccl2 mRNA in Müller cells inhibited the recruitment of both ED1-positive and IBA1-positive monocytes/microglia to the injured retina after BCL exposure. Third, our data showed that photoreceptor death was reduced after BCL when Ccl2 expression was inhibited by Ccl2 siRNA.

Previous investigators have theorized that Müller cells or retinal pigment epithelial cells (RPE) may be the source of chemokines that mediate neuroinflammation following light-induced degeneration [67],and several studies have shown that RPE cells *in vitro* express Ccl2 in response to stimulatory cytokines in the extracellular environment [68-72]. The present study is the first, to our knowledge, to directly confirm that Müller cells guide monocyte/microglia recruitment in the retina through the expression of Ccl2 mRNA, and that such expression exacerbates photoreceptor death following the initial damaging-light stimulus. This is consistent with our previous investigation, which found that Müller cells express Ccl2 in spatiotemporal correlation with the recruitment of ED1-positive monocytes and photoreceptor death following BCL exposure [55].

Our data point to a crucial role for chemokines in the propagation of local neuroinflammatory responses driven by the neural retina. Ccl2 is a strong chemoattractant and activator of monocytes [46] and microglia [47]*in vitro*, and is induced in the CNS in a range of pathologies (reviewed in [42]). Our data indicate that Ccl2 upregulation by Müller cells promotes the recruitment of two monocyte/microglia populations immunoreactive for the markers ED1 and IBA1 in the retina following BCL exposure [65]. First are parenchymal microglia immunoreactive for IBA1, which infiltrate the OPL and ONL after BCL [63,65]. Second, there is modulation of ED1+/IBA1+ nuclei recruited from the retinal and choroidal blood supplies, which is consistent with the markers, morphology, and distribution of bone-marrow-derived ‘hematogenus’ monocytes [65,66,73]. These findings are supported by a previous study in the CNS using Ccr2-knockout mice subjected to partial sciatic nerve ligation, which showed that the Ccr2 chemokine receptor, of which Ccl2 is a known ligand [74], mediates the recruitment of both hematogenous and resident microglia/monocytes immunoreactive for IBA1 [75].

Because both bone-marrow and resident microglia/monocytes are implicated in the clearance of debris and dead photoreceptors after injury [66], the expression of Ccl2 by Müller cells may promote homeostasis and recovery through efficient recruitment and activation of phagocytes to sites of photoreceptor degeneration. However, given that we found a decrease in photoreceptor apoptosis and expression of AP-1 following suppression of Ccl2 by siRNA, the secretion of Ccl2 by Müller cells may be a maladapted process, which is prone to eliciting exaggerated and damaging microglial responses. A number of studies have shown that microglial activation and aggregation exacerbates photoreceptor degeneration in the light-damage model [32,33], whereas activated microglia induce apoptosis of cultured photoreceptors through the secretion of cytotoxic factors *in vitro*[22]. Moreover, the introduction of synthetic Ccl2 to cultured microglial cells or monocytes has been shown to promote their activation and cytotoxicity toward co-cultured photoreceptors and RPE cells [47,76]. The signaling events that govern the synthesis of Ccl2 by Müller cells are unknown, although upregulation of Ccl2 may be stimulated as a result of local photoreceptor death, because increased levels of Ccl2 in Müller cells correlates spatially with the localization of light-induced photoreceptor apoptosis, as shown in our previous investigation [55]. Alternatively, or perhaps concurrently, Ccl2 synthesis may be stimulated by the presence of cytokines in the extracellular environment, such as IL-1β, IL-7, and TNF-α [68,71,77], following BCL exposure.

Our findings are consistent with other studies that have characterized Ccl2 as a non-redundant factor in the guidance of microglia/monocytes in a variety of degenerative models. In the retina, an investigation in experimental retinal detachment using Ccl2^−^/^−^ mice and Ccl2-specific antibody neutralization noted a substantial decrease in the recruitment of parenchymal microglia to the ONL following detachment, in conjunction with reduced photoreceptor death [47]. Deficiencies in monocyte recruitment have also been reported after Ccl2 inhibition in other models such as skin inflammation [78], thioglycollate challenge [79], experimental autoimmune encephalomyelitis [80], pulmonary granuloma [79], and peripheral endotoxin insult [81]. Despite this, a previous investigation did not observe modulation in a population of F4/80-positive macrophages in the subretinal space following light-induced damage to Ccl2^−^/^−^ mice [82]. As discussed in our previous investigation, however [55], the authors in that investigation did not quantify those cells, nor did they assess the distribution of other microglial markers such as ED1 and IBA1.

### Relevance to human retinal dystrophies

Exposure to bright continuous light in rats has been used to model retinal degeneration for over 40 years [83,84]. Several lines of evidence also indicate that light damage is a useful model of AMD [56,85-87]. This model, like the established laser-induced model of neovascular AMD, uses an acute damaging stimulus to evoke site-specific AMD-like retinal degeneration. Although the rat retina lacks a macula and *fovea centralis*, it includes an homologous feature, the *area centralis*, in superiotemporal retina [88-90]. Previous studies have identified the focal degeneration of photoreceptors and RPE cells and associated changes to the blood–retinal barrier as being localized to the *area centralis*, thus mimicking many of the histopathological aspects of advanced ‘dry’ AMD [56,85-87].

Recruitment of monocytes/microglia has been associated with the progression and severity of AMD pathology for many years [2,12-15], while several investigations have shown that microglial attenuation reduces lesion size in the laser-induced model of neovascular ‘wet’ AMD [91-93]. Retinas from human donors show increased expression for Ccl2 in all forms of AMD [94], while increased levels of Ccl2 protein have been detected in aqueous humor samples taken from patients in advanced stages of ‘wet’ and ‘dry’ AMD [95,96]. Increased *Cc2* expression has also been described in the retinas of aged (20-month-old) mice, compared with young (3-month-old) mice [97]. Moreover, studies in experimental laser-induced choroidal neovascularization (CNV) have shown that ablation of either *Ccl2* or the receptor *Ccr2* inhibits the infiltration of monocytes/microglia and reduces lesion size following CNV [98,99]. Conversely, it has been previously suggested that aging Ccl2^−^/^−^ Ccr2^−^/^−^ mice develop AMD-like retinal degeneration [100,101], indicating that a degree of Ccl2 signaling is also required for homeostasis, although the AMD-like phenotype in the knockout has been questioned [98].

siRNA-mediated gene therapy is considered to have therapeutic potential in knocking down deleterious genes in various human pathologies (reviewed in [102,103]). Our investigation is the first to show that monocyte recruitment, and in turn photoreceptor death, may be modified in the retina by siRNA-mediated suppression of Ccl2 *in vivo* in the CNS. Previous studies in AMD have shown that intravitreally injected siRNA targeting vascular endothelia growth factor ameliorates retinal degeneration in experimental CNV [104,105], and has also been the basis for several clinical trials [106]. However, unlike the current investigation, these early studies used ‘naked’ unmodified siRNA molecules, which are now known to produce non-specific effects via Toll-like receptor 3 signaling in the retina [106]. Nevertheless, modulation of Ccl2 expression using appropriately targeted RNAi may provide a powerful means to control excessive microglial recruitment and activation in retinal dystrophies such as AMD.

## Conclusion

Targeted suppression of Ccl2 in Müller cells by siRNA inhibits recruitment of monocytes/microglia and ameliorates apoptosis of photoreceptors following BCL exposure. Although the recruitment of phagocytes by Ccl2 may be geared toward beneficial function after retinal injury, our data suggest that robust Ccl2 secretion by Müller cells leads to an excessive aggregation of activated monocytes/microglia, leading to further photoreceptor degeneration. We therefore suggest that therapeutic attenuation of microglial recruitment using RNAi may be a useful strategy to control detrimental immune responses in the retina, which has relevance for the treatment of human pathologies such as AMD.

## Competing interests

The authors declare that they have no competing interests.

## Authors’ contributions

MVR designed and conducted the experiments, conducted the analysis, and wrote the paper; RCN designed and conducted the experiments; and JMP designed the experiments, and wrote the paper. All authors read and approved the final manuscript.
